# Is qPCR a Reliable Indicator of Cyanotoxin Risk in Freshwater?

**DOI:** 10.3390/toxins8060172

**Published:** 2016-06-07

**Authors:** Ana Beatriz F. Pacheco, Iame A. Guedes, Sandra M.F.O. Azevedo

**Affiliations:** Carlos Chagas Filho Biophysics Institute, Federal University of Rio de Janeiro, Rio de Janeiro 21949-902, Brazil; iameguedes@gmail.com (I.A.G.), sazevedo@biof.ufrj.br (S.M.F.O.A.)

**Keywords:** cyanobacteria, microcystin, saxitoxin, cylindrospermopsin, bloom

## Abstract

The wide distribution of cyanobacteria in aquatic environments leads to the risk of water contamination by cyanotoxins, which generate environmental and public health issues. Measurements of cell densities or pigment contents allow both the early detection of cellular growth and bloom monitoring, but these methods are not sufficiently accurate to predict actual cyanobacterial risk. To quantify cyanotoxins, analytical methods are considered the gold standards, but they are laborious, expensive, time-consuming and available in a limited number of laboratories. In cyanobacterial species with toxic potential, cyanotoxin production is restricted to some strains, and blooms can contain varying proportions of both toxic and non-toxic cells, which are morphologically indistinguishable. The sequencing of cyanobacterial genomes led to the description of gene clusters responsible for cyanotoxin production, which paved the way for the use of these genes as targets for PCR and then quantitative PCR (qPCR). Thus, the quantification of cyanotoxin genes appeared as a new method for estimating the potential toxicity of blooms. This raises a question concerning whether qPCR-based methods would be a reliable indicator of toxin concentration in the environment. Here, we review studies that report the parallel detection of microcystin genes and microcystin concentrations in natural populations and also a smaller number of studies dedicated to cylindrospermopsin and saxitoxin. We discuss the possible issues associated with the contradictory findings reported to date, present methodological limitations and consider the use of qPCR as an indicator of cyanotoxin risk.

## 1. Introduction

After at least four decades of intensive studies on the occurrence of toxic cyanobacteria in different countries and environments, the primary causes and consequences of cyanobacteria dominance in different water bodies, the effects of cyanotoxins in various cell systems, in addition to the physiological effects and molecular synthesis of some cyanotoxins are already known. However, some challenges are still open, and one that is important for management and public health decisions is how to provide an effective early warning system for the development of potentially toxic blooms.

The WHO guideline Toxic Cyanobacteria in Water, as edited by Chorus and Bartram [1], already emphasized this topic and presented some options for establishing cyanobacteria and cyanotoxin-monitoring programs. Since that time, progress in the methods that are used to detect and quantify cyanotoxins in different matrixes has been made, and studies using molecular tools appeared as a promising option to improve these analyses and simultaneously make them faster and less expensive, complementing other risk assessment tools for monitoring aquatic environments impacted by cyanobacterial blooms.

Almost 20 years later, we believe that there is already enough accumulated information to allow us to critically analyze the actual usefulness of molecular tools to estimate the risk of exposure to cyanotoxins for biota and the public health. This review focuses on the use of quantitative real-time PCR (qPCR), a method widely used for quantifying genes of interest in microbial communities. In the last few years, the application of qPCR is increasing as a tool to quantify cyanotoxin genes in bloom samples. To discuss these data, we included information here that is available for the three primary freshwater cyanotoxins, namely microcystins, cylindrospermopsin and saxitoxins. We selected studies that used qPCR for the quantification of cyanotoxin genes in environmental samples and also reported cyanotoxin concentrations, thus providing a chance to evaluate whether qPCR would be a reliable indicator of bloom toxicity. We discuss the possible issues associated with the contradictory findings reported to date, present methodological limitations and consider the usefulness of qPCR as a complementary tool for early monitoring of toxic cyanobacterial blooms.

### 1.1. Traditional Methods for Cyanotoxin Detection

Many different approaches are currently used to monitor toxic cyanobacterial blooms. The early detection of cyanobacteria by using cell densities to represent cyanobacterial biohazards is usually performed by chlorophyll-a measurements or microscopy analysis. However, these approaches do not differentiate toxin-producing from non-toxin-producing strains. The determination of the cyanotoxin exposure risk depends on the direct quantification of cyanotoxins with biochemical or immunological assays, like the protein phosphatase inhibition assay (PPIA) and the enzyme-linked immunosorbent assay (ELISA), as well as analytical methods, such as high-performance liquid chromatography (HPLC) and liquid chromatography-mass spectrometry (LC-MS/MS).

Although they are considered the gold standards for toxin measurement, each of these methods has advantages and limitations. Colorimetric or fluorometric PPIAs are sensitive, cost effective and can be used for the routine screening of microcystin (MC) concentration, but since they are based on enzymatic activity inhibition, they are not specific. ELISAs, either based on polyclonal or monoclonal antibodies, are highly sensitive and specific, but expensive. Furthermore, in the case of MCs, the existence of numerous variants and the diverse levels of cross reactivity in these assays can lead to underestimation of some variants. Considering analytical methods, HPLC coupled with a photodiode-array detector for UV absorbance is commonly used for analyzing MC and cylindrospermopsin. To analyze saxitoxins, a fluorometric detector is necessary either for pre-column derivatization or post-derivatization methods. Despite its sensitivity and applicability to different cyanotoxins, HPLC methods are still expensive and dependent on the availability of analytical standards and well-trained technicians. Furthermore, the identification based on absorption profiles usually cannot distinguish cyanotoxin variants and is subject to interferences from other analytes. Chromatographic separation coupled with mass spectrometry (LC/MS) is a more advanced and powerful technique that enables the quantification of cyanotoxins with high sensitivity and the identification of variants in a mixture. Based on characteristic mass spectra of toxin-derived ions (as compared to a standard mass spectrum), it is highly specific and can be further improved by performing tandem MS. LC-MS/MS detection generates fragmentation patterns that can be used to identify unknown toxins in field samples. However, similar limitations observed for HPLC methods related to cost, standards availability and specific technical expertise are also observed.

Besides, all of these biochemical and analytical methods are to some degree affected by sample matrix interferences, which further complicate their application to environmental samples. In the case of MC, these methodologies usually use methanol cell extracts and measure only the free MC, but it has been shown that a significant proportion of MC is bound to proteins and is therefore neglected in these protocols [2]. Although this fact cannot be overlooked when studying the effect of environmental factors on toxin production, from a toxicological point of view, this may not be critical, since MC binds irreversibly to proteins, and this fraction probably does not contribute for the overall toxicity of MC-producing cyanobacteria. Besides the aspects cited here, a more thorough discussion of the pros and cons of these methods can be found in recent reviews [3,4,5].

What is usually reported in monitoring studies is a combination of more than one approach to support quantitative data on cyanotoxin concentrations. However, in general, these alternative methods are laborious, expensive, time-consuming and available in a limited number of laboratories. These limitations are especially concerning for regions where the potential exposure risk is higher because of inefficient water treatment systems and/or unsafe sanitation conditions. 

In addition, the toxic profile of a bloom can be unknown or the blooms can produce more than one cyanotoxin [6,7,8,9,10,11], and the majority of chemical methods that are used to analyze cyanotoxins generally detects only one type of toxin at a time. Additionally, the quantification of toxins is only possible when they are already present in the water at concentrations above a certain detection limit. The measured concentration reflects the situation in a water body at a specific moment, but this concentration can change rapidly because of diffusion (especially when the toxin is released), cell lysis, continuous toxin synthesis or variable toxin stability. 

Thus, to substitute or complement traditional methods, an ideal new technology for cyanotoxin detection would be simple, fast, sensitive and cost-effective and, thus, applicable to the continuous monitoring of toxic cyanobacterial blooms, even over short-term intervals. Additionally, the ideal method would have a predictive value, serving in the early detection of the potential toxicity of a bloom and providing rapid risk assessment to prevent more serious consequences. In principle, molecular assays based on PCR could meet these requirements.

### 1.2. Molecular Tests for Detecting Toxic Cyanobacteria 

Attempts to use molecular tests to detect toxic cyanobacteria were made even before the description of the genetic loci encoding enzymes that are responsible for cyanotoxin synthesis. In early studies, polymorphisms within the 16S rRNA gene, internal transcribed spacers (ITS) or phycocyanin loci were tested for the detection and differentiation of potentially toxic strains (in *Microcystis*, *Anabaena* and *Nodularia*) [12,13,14,15]. Although the polymorphisms in these loci were useful for differentiating genera or strains, no general pattern for distinguishing toxic from non-toxic strains was revealed, which prevented the use of these approaches for discriminating among toxic genotypes [15].

## 2. Identification of Gene Clusters Encoding the Enzymes Responsible for Cyanotoxin Synthesis

### 2.1. Microcystin 

The MC biosynthesis gene cluster (*mcy*) was first described in two strains of *Microcystis aeruginosa* [16,17]. This cluster spans 55 kb, including 10 open reading frames (ORFs) that are organized into two operons *(mcyA-C* and *mcyD-J)*, and they are transcribed in the opposite direction from a central regulatory region. These genes encode large multifunctional enzymes that are involved in the biosynthesis of this cyclic peptide, that act as non-ribosomal peptide synthetases (NRPS) or polyketide-synthases (PKS) (McyA-E, *G*), tailoring enzymes (McyJ, McyF and McyI) and one protein that was designated as a transporter (McyH).

In addition to *Microcystis*, MCs are predominantly produced by *Anabaena* and *Planktothrix*, and the *mcy* gene cluster was also later characterized for these genera [18,19]. A comparative analysis of the *mcy* gene cluster revealed the genetic differences among these genera, such as the gene arrangement within the operons and the presence or absence of certain genes that encode tailoring enzymes [20]. MC production was later described in freshwater strains of the *Fischerella* and *Phormidium* genera [21,22].

Phylogeny-based studies indicated that the *mcy* cluster is an ancient trait in cyanobacteria, and its current sporadic distribution in just a few genera is thought to be the result of repeated loss during evolution [23]. This idea also explains the coexistence of both toxic and nontoxic strains in MC-producing genera. Accordingly, *mcy* genes were originally present in these genera, and nontoxic strains result from the partial or total loss of the toxin cluster [24,25]. MC genes can also be inactivated by point mutations or insertion sequences [26]. Thus, nontoxic strains may retain part of the *mcy* cluster to a greater or lesser extent.

The existence of numerous MC variants is related to the evolution of microcystin synthetase genes that results in altered enzymatic activities. Genetic variations in *mcy* sequences can result from point mutations and recombination events, and in the latter case, they are related to the lateral transfer of *mcy* gene parts between the strains within a genus [27,28,29].

### 2.2. Cylindrospermopsin

The characterization of part of the gene cluster that biosynthesizes the alkaloid cylindrospermopsin (*cyr*) was first described in *Cylindrospermopsis raciborskii* and *Aphanizomenon ovalisporum* (*aoa* genes) [30,31]. The complete cluster was identified in *C. raciborskii* [32]. The cluster spans 43 kb, with 15 ORFs encoding biosynthetic enzymes, including amidino transferase (CyrA), PKS and NRPS activities (CyrB-F) and enzymes responsible for uracil ring formation (CyrG and CyrH), as well as tailoring functions (CyrI, CyrJ and CyrN). This cluster also contains genes that encode proteins related to the regulation (CyrO), transposition (CyrL and CyrM) and export of cylindrospermopsin (CyrK).

Cylindrospermopsin (CYN) is produced by strains of other filamentous cyanobacteria, including *Oscillatoria* sp., *Anabaena bergii*, *Anabaena lapponica*, *Umezkia nathans*, *Aphanizomenon flos-aquae, Raphidiopsis curvata*, *Lyngbya wollei* [20,33] and *Raphidiopsis mediterranea* [34]. Among these genera, similar *cyr* gene clusters have been described for *Aphanizomenon* sp. [35], *Oscillatoria* sp. [36] and *R. curvata* [37]. In comparing the structural organization of *cyr* clusters from CYN producers, the gene organization is similar in Nostocales, although there are rearrangements of conserved sections, and more divergent in *Oscillatoria* sp. Accordingly, the sequence similarity of *cyr* genes is equal to or higher than that of 16S rRNA genes in Nostocales, and lower for *Oscillatoria* sp. [37]. Rearrangements in parts of the *cyr* cluster may occur at the intragenome or intergenome levels, and in the intergenome case, these rearrangements are linked to the horizontal transfer of *cyr* genes [35,37]. Horizontal transfer is also a possible explanation for the sporadic distribution of *cyr* gene clusters in the phylogenetic tree of cyanobacteria, as further supported by the high sequence conservation, atypical GC content of the *cyr* gene cluster and evidence of transposition [35]. Alternatively, the high sequence conservation of CYN genes may indicate an ancient origin and the subsequent repeated loss of parts or the entire cluster. These events would explain the co-occurrence of CYN-producing and non-producing strains within the same species.

### 2.3. Saxitoxin

The first description of the saxitoxin (STX) biosynthesis gene cluster (*sxt*) was made in *C. raciborskii* T3 [38]. In this strain, the *sxt* gene cluster covers approximately 35 kb, encoding 31 ORFs. The larger gene in this cluster is *sxtA*, which encodes a multifunctional enzyme that initiates STX synthesis. Subsequent reactions during the biosynthesis of the alkaloid involve the enzymatic activities of transferases, hydroxylases, hydrolases, reductases and cyclases. The synthesis of STX analogues, such as hydroxylated (neoSTX), decarbamoylated (dcSTX) and *O*- and *N*-sulfurylated toxins, is performed by tailoring enzymes that introduce various functional groups. ORFs coding for transposases, regulators and a few ORFs with unknown functions are also included in the *sxt* clusters. The *sxtF* and *sxtM* genes encode proteins that are similar to sodium-driven multidrug and toxic compound extrusion proteins, and their products are likely involved in the export of STX from *C. raciborskii*.

The strains of many other freshwater species of cyanobacteria, such as *A. circinalis*, *Aphanizomenon* sp., *Aphanizomenon gracile*, *Aphanizomenon issatschenkoi*, *Raphidiopsis brookii*, *Lyngbya wollei*, *Planktothrix* sp., *Scytonema*, *Geitlerinema amphibium*, *Geitlerinema lemmermannii*, *Cylindrospermum stagnale* and *Phormidium uncinatum*, can also produce STXs [20,39,40,41]. Homologous *sxt* gene clusters have been identified in strains of some of the following species: *A. circinalis* AWQC131C, *Aphanizomenon* sp. NH-5 [42], *R. brookii* D9 [43] and *L. wollei* [44]. *R. brookii* presents the smallest *sxt* gene cluster, with 25.7 kb and 24 ORFs. Twenty of these ORFs are shared with *C. raciborskii*, constituting the minimum gene set for STX biosynthesis. *L. wollei* has the largest (36 kb) and most divergent *sxt* cluster compared to Nostocales, which contains unique genes and a distinct gene organization. Thus, different genera share a core set of *sxt* genes, and they differ in the presence or absence of certain genes; their organization and these differences are reflected in their toxic profiles [39]. 

A comparative analysis of *sxt* clusters in five genera showed high conservation as the result of strong stabilizing selection. The loss and rearrangement of genes led to diverse gene configurations that correspond to species phylogeny. This finding indicates that this cluster likely had a single ancient origin in cyanobacteria and has been vertically inherited [39,45]. The sporadic distribution of STX producers in cyanobacteria would result from repeated gene loss events. However, it is also postulated that a subset of *sxt* genes was acquired by horizontal transfer from cyanobacterial sources or the lack thereof [45].

## 3. qPCR for Cyanotoxins

**Figure 1:**
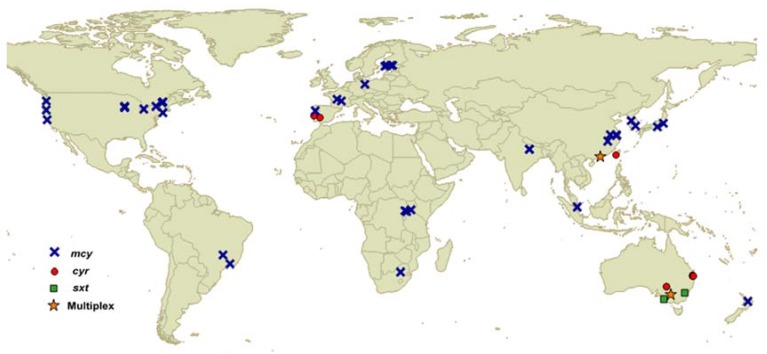
Geographic distribution of studies using qPCR for cyanotoxin genes in environmental samples. Map from the public domain map dataset Natural Earth (www.naturalearthdata.com).

**Figure 2:**
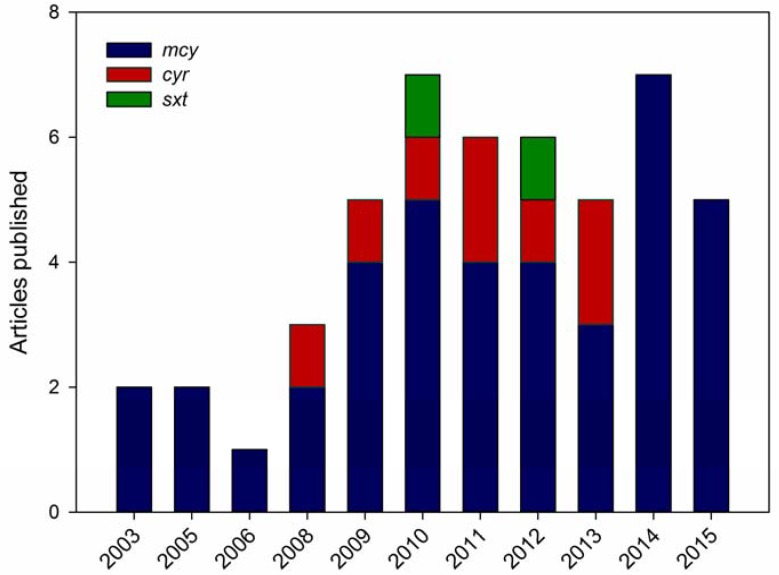
Chronological overview of literature available on qPCR for microcystin (*mcy*), saxitoxin (*sxt*) and cylindrospermopsin (*cyr*) gene detection in environmental samples.

The description of the biosynthesis gene clusters for cyanotoxins allowed for the development of PCR assays to detect sequence targets within selected genes, which served to identify potentially toxic strains. These assays were soon adapted for a qPCR format, enabling the quantification of toxin genes. This tool had an expected application for monitoring toxic blooms and also for studying the dynamics of fluctuations in potentially toxic to non-toxic genotypes in response to environmental factors. A methodological description of the qPCR method is not included here, but can be found in Martins and Vasconcelos [46], where its use in studying cyanobacterial population dynamics is also described, and in Kim *et al.* [47], where its application for quantitatively investigating environmental microbial communities is discussed. Here, we focus on the use of qPCR to assess the toxicity of cyanobacterial blooms that produce MC, CYN or STX, since these toxins are prevalent in freshwater and have been addressed with molecular methods. The geographical distribution of the studied environments is represented in Figure 1. A chronological overview of the literature available on qPCR for these cyanotoxins is shown in Figure 2.

### 3.1. qPCR for Detecting Microcystin Genes

Pioneer studies that used qPCR to quantify MC-producing genotypes appeared in 2002 and 2003. The first attempt involved a Taq nuclease assay to detect microcystin synthetase genes (*mcyA* and *mcyB*) [48]. This assay was able to discriminate between the toxic and nontoxic strains of *Microcystis*; additionally, a preliminary test was made with cyanobacterial environmental samples, but no data on parallel MC quantification were reported. 

Later, Kurmayer *et al.* [49] proposed a different method for quantifying toxic genotypes by combining primers to quantify the total *Microcystis* cells (with the intergenic spacer region of the phycocyanin operon as the target) and MC-producing genotypes (*mcyB* gene as the target). In this study, relative quantification was based on serial dilutions of template DNA and not on a conventional qPCR assay. In using this tool, the authors concluded that higher *mcyB*-to-phycocyanin ratios corresponded to higher MC cell quotas and that variations in the proportion of MC-producing genotypes could explain the MC net production in *Microcystis* colonies of different sizes.

During the same year, Kurmayer and Kutzenberger [50] developed a Taq nuclease assay that also targeted the phycocyanin locus and the *mcyB* gene, but by using new primes/probes. The assay was validated with laboratory strains of *Microcystis* and also with samples from *Microcystis* blooms. This study concluded that cell quantification by PCR correlated significantly with cell numbers as determined by microscopic counting, and the investigators applied this method to following seasonal shifts in MC genotype proportions in a lake. However, toxin concentrations were not measured, and correlations between *mcyB* quantification and toxicity were not evaluated.

Also in 2003, Vaitomaa *et al.* [51] applied qPCR by using a set of primers to target *mcyE*, which was designed to distinguish *Microcystis* from *Anabaena* strains. The *mcyE* copy numbers correlated positively with the MC concentrations, and it was postulated that this method could be valuable for monitoring hepatotoxic blooms. However, it should be noted that a positive correlation between the MC concentration and *mcyE* copy number depended on whether the *mcyE* data were considered individually for *Microcystis* or *Anabaena*, or in combination (as the sum of *mcyE* copy numbers for both), and it also depended on whether the samples from the two lakes were considered individually or in combination.

From that year on, a number of studies that combined qPCR and MC quantification to analyze toxic cyanobacterial blooms appeared (Figure 2), and these tools are still proposed for risk assessment. These studies are shown in Table 1. This set of papers was the result of searches that were performed in PubMed, Science Direct and Google Scholar using the keywords “microcystin” and “qPCR”, and some papers were also recovered from the reference list of other studies. Studies dating from 2003 to 2015 are listed. Key aspects related to the nature of the studied samples, PCR design and the possible correlation between the MC concentrations and qPCR results or proxy parameters, such as chlorophyll-a and cell density, are included. In gathering these data, our primary intent was to estimate the degree to which the *mcy* gene copy numbers, as assessed by qPCR, correlated positively with the MC concentration. The sample fraction used for MC extraction and the detection method or the MC concentration were also listed to investigate if these factors would influence the correlation between qPCR results and toxin quantification. The target *mcy* gene that was selected for qPCR was included to provide an overview of the most common assay designs.

From the studies considered here, 22 reported a consistent positive correlation between the MC concentration and *mcy* gene copy number, and this correlation was not observed in 11 reports. Unfortunately, some studies were inconclusive in this respect, because this correlation was not tested [49,52,53,54]. There were also some cases in which the *mcy* copy numbers were determined, but the MC concentration was reported as the cell quota; therefore, the possible correlation could not be evaluated. In considering the studies that tested the correlation between the MC concentration and *mcy* gene quantification, we asked if certain methodological factors could influence the results. 

The most commonly-used target *mcy* genes were *mcyA*, *mcyB*, *mcyD* and *mcyE*. Primers are described for different regions of these genes, and they are generalists for any MC producer or specific for *Microcystis*. In approximately 80% of the cases in which *mcyE* was quantified, positive correlations were found with the MC content. For the other cited *mcy* genes, this correlation was found in approximately 60% of the cases. In principle, any *mcy* gene would be equally reliable for evaluating the presence of an *mcy* cluster and the number of toxic cells, because they are single copy genes. Even in studies that measured more than an *mcy* gene simultaneously, quantification resulted in similar patterns, but did not match completely for different *mcy* genes [55,56]. This finding can be attributed to different primer efficiencies when targeting *mcy* genes, or because of the presence of incomplete *mcy* clusters. In more recent studies, there is a tendency to choose target genes that code for enzymes that are involved in the first steps of cyanotoxin biosynthesis, with the aim of increasing reliability, avoiding genes that code for tailoring enzymes.

Considering the sample fraction from which MC was obtained, 60% of these studies used particulate material, and almost 40% measured the total MC (only one study measured dissolved MC). The quantification of *mcy* gene copies correlated better with MC concentrations when the total toxin concentration was considered (in 80% of the cases) than when only the particulate fractions were used (in 60% of the cases). Although MC is an intracellular compound, it can be released in water by cell lysis, and it can then be degraded by heterotrophic bacteria or adsorb to particulate matter. Thus, its extracellular concentrations are usually low [85]. However, from a risk assessment perspective, it is important to consider the total MC once significant concentrations can be found dissolved in water. This is especially true under conditions involving some mitigation actions to reduce cyanobacterial bloom density, which can include chemical control and/or the natural senescence of a bloom [54,83].

In relation to the method used for MC quantification, 32% of reports considered in our search used ELISA; 26% used HPLC; 18% quantified the MC by PPIA; 13% used LC-MS/MS; and 11% used more than one method. MC quantification by ELISA seems to correlate better with the *mcy* gene content because a positive correlation was found in all studies in which the immunoassay was used. When other methods were applied for toxin quantification, the percentage of studies that reported a positive correlation was lower (63% for HPLC, 50% for LC-MS/MS, 29% for PPIA). However, there is no consensus about which analytical method can be considered the standard for determining the MC concentration, because they vary in selectivity and sensitivity. Therefore, it is not possible to conclude that ELISA would be better for MC quantification in combination with *mcy* gene copy numbers. In fact, LC-MS/MS has been increasingly applied to quantify MC because of its superior specificity.

In summary, although methodological factors may favor finding a positive correlation between *mcy* qPCR data and MC contents, from the examination of this limited number of studies, it is not possible to conclude that a single technical aspect could be critical. 

Due to the fact that approximately one-third of the available reports did not identify this type of correlation, a question about the actual utility of qPCR for the risk assessment of toxic cyanobacterial blooms naturally emerges. As more studies appear, this conclusion can be re-evaluated, but the actual picture does not justify the conclusions found in many papers regarding the promise of qPCR for monitoring bloom toxicity [52,60,68,74,75,78]. 

However, for the same set of studies listed in Table 1, when the correlation between the MC concentration and chlorophyll-a or number of cyanobacterial cells was tested, it was positive in 84% of the cases. Thus, the degree to which qPCR is advantageous in comparison with these traditional monitoring approaches should be questioned during risk assessments of cyanobacterial blooms.

#### qPCR for Determining the Microcystin Toxic Genotype Proportion

**Figure 3:**
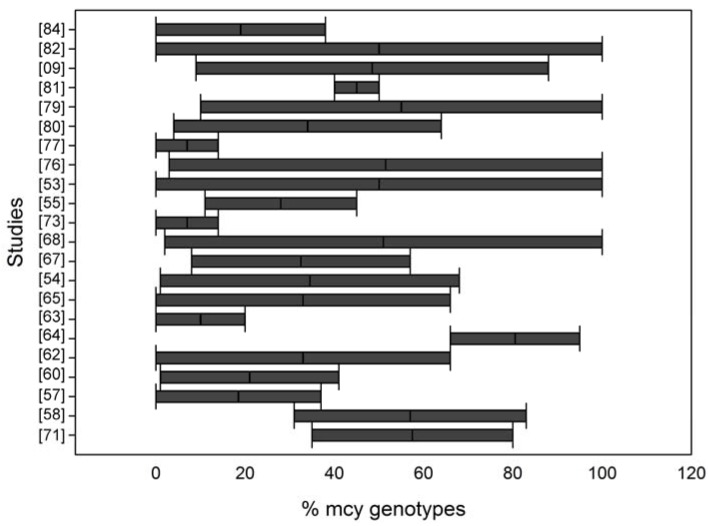
Comparison of the reported values for the percentage of potentially MC-producing genotypes in *Microcystis* blooms.

In some studies, the *mcy* copy number was directly related to MC concentrations, while in others, qPCR was designed to quantify both *mcy* genes and a housekeeping gene (16S rRNA or *cpc*) to account for the total cyanobacteria or *Microcystis* abundance, and the percentage of toxic genotypes is reported (Figure 3). Thus, a possible correlation between the percentage of toxic genotypes and MC concentrations was tested. The results are contradictory in this regard. In some cases, the MC content showed a clear correlation with the proportion of potentially toxic cells [9,57,60,65,76,84]. However, in other studies, this finding was not observed [55,62,63,64,67,68,73,79,81,82]. In some cases, even if the *mcy* copy numbers alone showed a significant positive correlation with the MC concentration, the percentage of toxigenic *Microcystis* did not show this correlation. 

The translation of gene copy numbers into cyanobacterial cell numbers is prone to errors, according to numerous studies. Toxin genes are present in single copies in the genome of sequenced strains. Consequently, in principle, the quantity of these genes should never outnumber the 16S rRNA gene (a multiple copy gene) count or the cell concentration, as determined by microscopy. However, several studies reported toxin gene numbers that were greater than the cell numbers [10,51,52,55,60,62,68,69,70,72,79,82]. When another gene is quantified to estimate the total cyanobacteria density (a 16S rRNA gene or *cpc* locus, for example), inconsistent results between the gene copy numbers and total cyanobacteria cell densities are commonly found [10,52,63,67,80,84]. This inconsistency results in a cumulative source of errors that is reflected in the *mcy*/16S rRNA gene ratio. 

There are multiple reasons for errors during the quantification of gene copy numbers by qPCR; there is natural variability in the gene copy numbers (16S rRNA) in the genomes of different species, polyploidy, the presence of an unknown number of target gene copies per cell because of ongoing chromosome replication, the presence of environmental DNA from dead cells that serve as PCR templates, a loss of cells when processing samples for DNA extraction, the incomplete recovery of DNA from cells, the presence of enzyme inhibitors or the differential selectivity of primers because of genetic variation in the target regions of different strains and species. All of these issues are more complicated in the case of 16S rRNA quantification because it is a multiple copy gene. However, errors in cyanobacterial cell counting by microscopy are also possible (particularly for filamentous cyanobacteria and for picoplanktonic species). Thus, when qPCR gene abundance is compared to cell counts, deviations from both approaches are expected. 

### 3.2. qPCR for Detecting Cylindrospermopsin Genes

In comparison with microcystin, the number of studies that apply qPCR to monitor CYN-producing blooms is reduced. These studies are listed in Table 2 and are briefly discussed below. 

In 2008, Rasmussen *et al.* [86] described the first qPCR assay for detecting CYN-producing cyanobacteria, more specifically, *C. raciborskii*. It consisted of a duplex Taq nuclease assay to target a *pks* genetic determinant (the *cyrC* gene, which was known at the time as the *C. raciborskii* homologue of the *A. ovalisporum aoaC* gene) and the *rpoC1* gene (a RNA polymerase gene). The *rpoC1* gene target sequence was specific for detecting *C. raciborskii*, and the toxin gene target sequence was common to diverse CYN-producing cyanobacteria. In environmental samples, the qPCR was specific, with positive results for all samples with *C. raciborskii* cell densities above 10^3^ cells mL^−1^. Although the detection of the toxin gene by qPCR was always consistent with positive results for CYN by LC-MS/MS, no correlation was observed between the *cyrC* copy numbers and toxin concentration. It was postulated that this finding occurred because a significant proportion of CYN is in the extracellular fraction and can be heterogeneously distributed in the water body, and during that study, only the intracellular fraction was considered.

In samples in which *C. raciborskii* was the only CYN-producing species, the *cyrC* and *rpoC1* copies were approximately 1:1, which was consistent with the fact that both genes are present in single copies in the *C. raciborskii* genome. This finding also indicated that all of the detected strains were potentially toxic. It was concluded that the method was sensitive and rapid for detecting potential CYN-producing cyanobacteria in field samples. 

A further evaluation of the usefulness of this qPCR assay was performed with samples from three subtropical reservoirs in Australia where *C. raciborskii* blooms were registered [87]. Relations between the total cyanobacteria, *C. raciborskii* cell density, CYN concentrations and qPCR results were analyzed. In addition to the duplex Taq nuclease assay developed by Rasmussen *et al.* [86], a single qPCR was performed to quantify the total cyanobacteria. Thus, the *cyrC* copy numbers were normalized by *C. raciborskii* cell counts, and this value was related to the CYN cell quotas derived from toxin quantification. A positive correlation between the *cyrC* cell quota and the CYN cell quota was found, and the authors concluded that a qPCR analysis of *cyrC* in combination with the *C. raciborskii* cell count could be used to estimate the intracellular CYN concentration in field samples. These investigators also found that the spatial and temporal variations in *cyrC* cell quotas in these reservoirs suggest that the toxicity of *C. raciborskii* blooms might result from the relative abundance of strains with different CYN cell quotas. However, it could also result from variations in the relative abundance of potentially toxic strains because the *cyrC* copy numbers were lower than the *rpoC1* copy numbers, at variable levels. However, in this study, qPCR was not effective as an alternative method for quantifying cyanobacteria, given that a poor linear relation was observed between the gene copy numbers (both *rpoC1* and 16S rRNA) and *C. raciborskii* cell concentration. 

A qPCR method that was similar to that reported by Orr *et al.* [87] was proposed by Moreira *et al.* [88], but no information about the correlation between the cell density and 16S rRNA or *rpoC1* copy numbers was reported, and validation with field samples and a comparison with toxin concentrations was not possible. 

The original idea of Rasmussen *et al.* [86] to apply qPCR to near real-time monitoring of CYN in the field was tested by Marbun *et al.* [89] in Taiwan’s reservoirs. This assay was tested with field samples, from which five samples were positive for *C. raciborskii* and two for CYN. In these cases, the microscopic cell counts and total toxin concentrations were in accordance with the qPCR results. All of the steps were performed on-site within 4 h after sampling, indicating that qPCR could be applied for the rapid on-site detection of toxic *C. raciborskii* in reservoirs.

A different qPCR assay using the *cyrA* gene as a target was able to detect different CYN-producing genera. It was included as part of a TaqMan-based multiplex qPCR assay reported by Al-Tebrineh *et al.* [92], which also included an estimation of total cyanobacteria through the quantification of 16S rRNA genes. This assay was applied to samples from a mixed cyanobacterial bloom [8]. The temporal variation of *cyrA* copy numbers over three sites indicated that CYN-producing cells initially dominated the bloom and then declined. The toxin concentration correlated positively with *cyrA* copy numbers.

A TaqMan-based qPCR assay for quantifying *A. ovalisporum* CYN production was developed by Campo *et al.* [90]. This assay was able to discriminate CYN-producing *A. ovalisporum* strains from other Nostocales, such as *C. raciborskii* and *A. bergii*. This approach was tested using field samples, for which the presence of CYN-producing *A. ovalisporum* was demonstrated. In three samples, the quantification of *cyrJ* gene copy numbers showed a positive correlation with extracellular CYN concentrations. The copy numbers of *rpoC1* were higher than the *cyrJ* copy numbers in one sample, suggesting the presence of non-toxic *A. ovalisporum* cells. In spite of the limited number of samples, the authors concluded that the qPCR assay was sensitive and specific for the quantification of potential CYN-producing *A. ovalisporum* in environmental samples.

In a recent study, qPCR was applied to track shifts in the proportion of toxic and nontoxic strains of *C. raciborskii* in response to nutrient availability during a bloom [91]. Upon testing the effects of different nutrient concentrations on toxicity, a high correlation was reported between the *cyrA*/16S rRNA ratio and the calculated CYN cell quotas, indicating that shifts in the relative proportion of toxic and non-toxic strains could be considered a major cause of variation in bloom toxicity.

Another application of qPCR for detecting CYN producers was reported as part of a multiplex reaction [56] (see the multiplex section below). In this case, the *cyrC* copy numbers were highly correlated to the CYN concentrations, *rpoC1* copy numbers and *Cylindrospermopsis* cell density, indicating that *C. raciborskii* was the CYN producer in these samples, and a high proportion of potentially toxic cells was present.

Given that the original proposal was intended to employ a qPCR assay to detect CYN-producing cyanobacteria, the primary objective was to use it as a molecular approach to estimate the potential toxicity of blooms rapidly. Thus, the critical factor in the usefulness of this method is whether the *cyr* gene copy numbers reflect the CYN concentrations in water samples. Most studies listed here have tested this possibility, and in some cases, a positive correlation was found between the toxin concentration and *cyr* gene copy number. However, it should be noted that this approach resulted from the use of different methodologies to quantify the CYN (Table 2). CYN is known to be released from cells, and a significant proportion can persist when dissolved in water [93,94]. Thus, to achieve an accurate estimation of toxin concentrations in water, it is necessary to combine the particulate and dissolved fractions or to convert the concentration to a cellular quota when only the intracellular fraction is analyzed. 

The combination of a *cyr* gene with a marker gene (*rpoC1*) to assign toxin production to a certain species was found to be useful for both *C. raciborskii* and *A. ovalisporum* blooms. Although a consistent correlation between the cell concentrations and the *rpoC1* gene copy number was not always met, the ratio between the *cyr* and *rpoC1* numbers could be a good indicator of the proportion of potentially toxic strains in the population. 

### 3.3. qPCR for Detecting Saxitoxin Genes

The characterization of the STX biosynthesis gene cluster in different species of cyanobacteria made it possible to develop a qPCR assay to quantify potentially STX-producing cells [95]. This assay was based on the detection of the *sxtA* gene. Primers were designed by considering the low variability of *sxtA* gene sequences in *A. circinalis* isolates. As an internal control, a pair of primers was included to target a 16S rRNA gene sequence that is conserved in all cyanobacteria. The *sxtA* primers were able to generate specific products from other STX-producing species (*L. wollei, Aphanizomenon* sp. and *C. raciborskii*), but qPCR resulted in lower amplification efficiencies and different melt curve profiles compared to the results for *A. circinalis*.

The *sxtA* qPCR assay was then applied to 13 samples of cyanobacterial blooms that were collected from diverse ecosystems in Australia (Table 3). A microscopy analysis revealed that *A. circinalis* was the dominant species in these samples. The STX concentrations determined by HPLC correlated positively with the *sxtA* copy numbers. The qPCR-inferred STX concentrations resulted in higher values than those that were directly measured by HPLC. Another discrepancy was observed in some samples in which the estimated cell density based on *sxtA* copy numbers gave higher values than microscopic cell counts. This study indicated that the *sxtA* copy numbers could be used to estimate the potential toxigenicity of toxic *A. circinalis* blooms. However, for other STX-producing cyanobacteria, this assay may require further optimization. Although this optimization can pose a problem when establishing a general method for detecting STX-producing cells, this sequence variability could be explored to develop a more discriminatory qPCR assay that would be able to distinguish STX-producing cyanobacterial species in environmental sample.

Later, the same group proposed another approach to *sxtA* detection as part of a multiplex qPCR assay for targeting the genes of several cyanotoxins, as described below in the multiplex qPCR section [96]. In this case, a new set of primers/probes for *sxtA* was designed on the basis of a target sequence that was conserved among four different genera. This assay was then applied to samples from mixed blooms that were occurring in an Australian river, in which both *A. circinalis* and *C. raciborskii* were reported [8]. The *sxtA* gene was detected in almost all samples, and the copy numbers indicated temporal variations in the amount of STX-producing cells. The qPCR results were consistent with the STX concentrations that were determined by ELISA.

It is evident from the data available in the literature that the use of qPCR to monitor STX-producing cyanobacteria in the environment is scarce. Although the *sxtA* copy numbers were shown to correlate with the toxin concentration, a greater number of studies is needed to evaluate the applicability of this method to monitor water samples, particularly in samples containing other species besides *A. circinalis.*

### 3.4. Multiplex qPCR for Cyanotoxins

Almost all studies that pertain to the molecular detection and quantification of cyanotoxin genes in environmental samples are focused on a single target gene and evaluate the potential toxicity of only one class of toxins. However, one species of cyanobacteria may produce different classes of cyanotoxins, and during cyanobacterial bloom events, a diverse composition of species with different toxic profiles can be found [6,7,8,10,56,64]. If the strength of qPCR is that it serves as a rapid and simple protocol to monitor water supplies for the presence of potentially toxic cyanobacteria, this method should ideally simultaneously detect and quantify the most common cyanotoxins. This motivation led to the development of multiplex qPCR assays. 

Multiplex qPCR is an effective solution to save time, samples and costs, but the design of a successful assay depends on the choice of compatible primers and probes and the optimization of reaction conditions to obtain the required sensitivity and specificity for each of the combined targets. This assay includes TaqMan probes that carry different reporters with distinct fluorescent spectra at the 5’ end and a quencher group at the 3’ end.

The first described multiplex qPCR for cyanotoxins targeted four different toxin biosynthesis gene clusters simultaneously, namely microcystin, nodularin, cylindrospermopsin and saxitoxin, as well as the 16S rRNA gene as an internal control [96]. The selected cyanotoxin targets were *mcyE/ndaF*, *cyrA* and *sxtA*, which were all based on sequences with at least 90% identity among the strains of different cyanobacteria genera. The specificity of the multiplex qPCR was validated by testing 51 toxic and non-toxic cyanobacterial strains. Once the proposed method was shown to be specific, sensitive and reliable, the next step was to apply it to environmental samples.

This strategy was soon used to evaluate the toxigenicity of mixed cyanobacterial blooms occurring in the Murray River, Australia [8]. *A. circinalis*, *M. flos-aquae* and *C. raciborskii* were detected by microscopic analyses and with samples collected from different sites that varied in their community composition and cyanotoxin profiles. Therefore, this study constituted an ideal situation to test the applicability of the multiplex assay. The toxicity assessment with ELISA revealed low STX concentrations in some samples and no toxin in others, low concentrations of CYL in all samples and no MC. A temporal analysis indicated that a bloom was initiated with a higher proportion of CYN-producing cells that were gradually substituted for STX-producing cyanobacteria. A positive correlation was observed between the toxin gene copy numbers and cyanotoxin concentrations as determined by ELISA. The authors concluded that the consistency between the qPCR data and the toxin concentrations for the three tested toxins supported the applicability of the multiplex assay to bloom risk assessment. This use of a single reaction to detect and quantify biosynthesis genes for the major cyanotoxins found in environmental samples allows for the characterization of toxigenic cyanobacterial assemblages, as well as the monitoring of dynamic changes in toxigenic profiles of complex blooms.

Multiplex qPCR-based monitoring was also performed in a reservoir in Macau, China, where abundant populations of *Microcystis* and *Cylindrospermopsis* were present and MC and CYL were detected some years before [56]. A total of 72 water samples were tested. The multiplex assay was designed to quantify the total *C. raciborskii* cells (*rpoC1* gene), CYN-producers in general (*cyrC* gene), total *Microcystis* (*Microcystis* 16S rRNA gene) and MC-producers (*mcyA/B/C/D/E/G/J* genes). This study reported the successful application of multiplex qPCR in water samples, confirming the value of this method for estimating CYN-associated risk, but also showing that in the case of *mcy* genes, the assessment of toxin levels by qPCR may be uncertain.

Although only a few studies have applied multiplex qPCR to environmental samples, the potential use of this method as a tool for monitoring water supplies was anticipated. This approach has many advantages for use in field sample analysis as follows: high sensitivity, broad dynamic range allowing the quantification of very scarce or highly abundant targets, a high throughput capacity, cost-effectiveness and fast results. The disadvantages of multiplex qPCR are related to its laborious optimization steps. These steps are required because of the competition between primers or between targets, cross-oligo interactions and difficulties in quantifying the gene targets in complex samples containing predominantly background DNA [96,97]. The determination of absolute 16S rRNA gene copy numbers can also be problematic with qPCR because of the variability in the number of these genetic loci in the genomes of different cyanobacteria species. Nevertheless, the relative quantification of multiple genes can be of special interest when monitoring spatial-temporal changes in the toxigenic composition of complex blooms.

### 3.5. Advantages of qPCR for Estimating Cyanotoxin Concentrations in the Environment 

Although we question the use of qPCR for estimating the toxicity of cyanobacterial blooms, this method is valuable for the study of toxic cyanobacteria population dynamics. Considering that qPCR is a fast, simple and cost-effective method and is easily applicable for the analysis of multiple samples, it is a suitable choice for exploring temporal and spatial variations in the relative abundance of toxic strains, improving our understanding of the dynamics of cyanobacterial blooms and their relations to environmental factors.

It is possible to further improve the simplicity and speed of the assay by implementing protocols that are compatible with crude cell extracts (which is not possible with other analytical methods) and with portable PCR equipment, configuring the assay to a near real-time detection test.

For the assessment of complex bloom samples, qPCR is extremely valuable because many cyanotoxin genes can be investigated simultaneously, while the analysis of different toxins by other analytical methods requires the use of different methodologies (LC-MS/MS or ELISA kits).

In addition, the combination of generalist and specific primers during qPCR permits the identification of the cyanobacteria genus or species responsible for producing the toxin in mixed blooms and to follow variations in their relative contributions over time or space.

Because of its high sensitivity (a detection of less than 10^2^ gene copies per mL), qPCR is advantageous over microscopic examination for detecting toxin-producing cyanobacteria when they are present in minor concentrations. For example, as reported by Lee *et al.* [83] in Lake Vancouver, although the *Microcystis* sp. abundance rarely exceeded one percent of the total cyanobacteria and was rarely detected in microscopic counts, the qPCR results indicated that the majority of the *Microcystis* population contained the *mcyE* gene, and the MC concentrations repeatedly exceeded WHO guidelines for drinking water. In addition, microscopy-based monitoring focuses on biomass increases as indicative of risk, but the MC content can be high even when the cyanobacterial abundance is low (for example, prior to bloom proliferation), and in this case, PCR can detect potentially toxic strains and can be important as an early monitoring tool.

### 3.6. Limitations of qPCR for Estimating Cyanotoxin Concentrations in the Environment

Since the development of early assays, qPCR was considered a promising tool for monitoring potential cyanotoxin producers in field samples, and the basic premise for its applicability is that a cyanotoxin gene copy number has a positive correlation with cyanotoxin concentrations in the samples.The result depends on accurate measurements of gene contents [47] and also on the method selected for cyanotoxin analysis [3,4]. In qPCR design, absolute quantification of a target gene is based on a standard curve relating known concentrations of a standard DNA to threshold cycle values. The calibrating DNA can be genomic DNA from an isolated strain, a plasmid carrying the target gene or purified amplicons. This curve is then used for extrapolating the concentration of the target sequence in an experimental sample. Absolute quantification based on this calibration assumes that both standard DNA and test samples are amplified with equal efficiency, which may not be true since environmental samples consist of a mix of DNA templates, abundant background DNA and may contain polymerase inhibitors. Depending on the nature of the sample, the extracted DNA can contain contaminants that result in PCR inhibition, affecting reproducibility and sensitivity, leading to false negative results. False negatives can be prevented by including universal targets as internal controls, such as rRNA genes, or by spiking standard targets in amplification reactions. These are possible causes of inconsistencies between cell counts and copy numbers of housekeeping genes. Another cause may be the incomplete recovery of DNA from environmental samples. The efficiency of DNA recovery can be different for different cyanobacterial species and also depends on the extraction method employed. 

Quantification can also be adversely affected by copy number variation of target genes or genomes per cell in different species present in environment samples. Toxin genes are present in single copies in the genome of sequenced strains; hence, the quantification of these genes should never outnumber the cell concentration, in principle. However, several studies reported toxin gene numbers that were greater than the cell counts. Possible reasons considered here included errors in cell counting or natural variation in the cellular copy numbers of target genes. In cases where 16S rRNA gene counts are included to estimate the number of cyanobacterial cells, this kind of error is magnified due to copy number variation. The copy number of the 16S rRNA gene varies between one and 15 per genome among bacteria [47]. Microbial communities are composed of diverse species with different numbers of 16S rRNA genes per genome, and the sequences of these multiple 16S rRNA genes on a genome can be distinct. Thus, these variations hinder the use of this gene to infer the abundance of cells. To overcome these problems, many studies use single copy genetic markers, such as the *rpoC1* gene or the *cpc* locus. However, these markers are still underrepresented in reference databases as compared to 16S rRNA gene sequences [47].

A qPCR assay depends on the choice of primers and probes to target the sequence of interest. First, the design of primers/probes is laborious, mainly for TaqMan assays and for multiplex applications. Primers/probes should ideally recognize target sequences in every toxic strain or species. However, because primer/probe design is based on the alignment of a limited number of reference sequences, it is not known which proportion of toxic cells will fail to be detected because of sequence variability in toxin genes. Thus, the use of generic primers/probes can lead to unequal amplification efficiencies for different species or genera, and this situation can result in the underestimation of toxic cells by qPCR. As the number of sequences in the databases increases, the design of primers/probes should be updated and optimized to improve coverage by detecting new variants of the target gene.

Another recognized problem is the presence of mutated, rearranged or partially deleted versions of toxin biosynthesis clusters in some strains and that the proportion of strains containing non-functional clusters in natural samples is not known. Because the target regions chosen in PCR assays correspond to very short sequences (approximately one hundred base pairs), they can result in an undetermined level of false positives. 

An important consideration is that the toxin gene abundance informs the investigator about the potential of the population for toxin production, and analytical methods, such as LC-MS/MS or ELISA, determine the actual toxin concentrations. Thus, incongruent results could be related to intrinsic variations in toxin production by different strains and/or regulated changes in toxin biosynthesis in response to environmental conditions. 

Another potential cause for disagreement between LC-MS/MS or ELISA results and qPCR is that the latter is sensitive enough to detect minor populations of potentially toxic cells, which do not produce toxin amounts above the limit of detection of the analytical methods. In addition, some toxin variants can be missed depending on the protocol for LC-MS/MS or the chosen ELISA kit. 

The choice of the sample fraction that is used when analyzing toxin concentrations is a highly variable issue in studies dealing with environmental samples, and they vary from intracellular (converted or not to a cell quota), particulate or extracellular material or else the total water sample. This variation makes the comparison of different studies and conclusions about correlations difficult for the quantification of toxin genes and toxin concentrations. Considering MC measurements, as indicated in Table 1, most studies analyze the particulate fraction, either alone or in combination with the dissolved content. However, in cyanobacterial cells, a large amount of MC is complexed with proteins and is lost in standard methanol extraction [2]. This is also a possible cause of discrepancy between the quantification of toxin genes and MC toxin concentrations.

The above-mentioned factors are the most commonly-recognized causes of inconsistency between toxin concentrations and qPCR results. It is clear that multiple technical and biological issues contribute to this situation, which limits the use of the molecular assay by itself for risk management at present. The general picture drawn from the literature is that traditional analytical methods and PCR should be combined to assess the presence of cyanotoxins in environmental samples.

## 4. Concluding Remarks

Despite its long history, the use of multidisciplinary approaches and the various methods already developed for cyanotoxin quantification, an effective tool for evaluating the risk of cyanotoxin exposure is still a challenge. The ideal goal would be a simple, cost-effective and fast method that was independent of direct cyanotoxin measurement. Over the last decade, qPCR has appeared as a promising tool to facilitate this task. Indeed, numerous studies supported the idea that qPCR results could be used to estimate the toxin contents in freshwater and contribute to rapid risk evaluation. However, as described here, there are also a considerable number of studies in which this conclusion was not made. 

Here, we consider possible reasons for such incongruent results that, in our view, hinder the use of qPCR to estimate the risk of exposure to cyanotoxins. Some points are related to qPCR technical limitations, particularly critical when environmental samples are concerned. These technical constraints preclude the use of gene copy numbers to determine the absolute abundance of cells, even for cell quantification. Another level of complexity refers to the variability of biological factors that affect toxin concentrations, a subject with intricacies that even a perfect qPCR assay will not overcome. Toxin concentrations depend on the cyanobacterial biomass abundance, the amount of toxic cells and the cell quota. In theory, qPCR could be used to estimate the total cyanobacterial biomass and the amount of potentially toxic cells, but it does not assess the toxin cell quota. The latter is influenced by environmental factors that may regulate toxin synthesis or can vary because of intrinsic differences in toxin production by different strains. Finally, once produced, the fate of the toxin is variable, and it can accumulate intracellularly (in a free form or bound to proteins), released or degraded. Thus, it is difficult to predict the concentration of bioavailable toxin from the assessment of potentially toxic cells. 

Monitoring cyanobacteria in natural communities still depends largely on microscopic counts and photosynthetic pigments (chlorophyll and phycocyanin) measurements to estimate biomass. These methods are good indicators of the potential risk of cyanotoxin exposure and are especially useful in remote regions where the use of molecular tools is still limited by cost and/or lack of expertise. This is noticeable from the numerous studies cited here that reported a positive correlation between the MC concentration and chlorophyll-a or number of cyanobacterial cells. Of course, this analysis can be improved by assessing the presence of potentially toxic strains, which can be rapidly revealed by conventional or qPCR, ideally with multiplex assays that are able to detect any cyanotoxin class. However, the confirmation of the toxin presence and the determination of its concentration still relies on biochemical or chemical analytical methods, such as ELISA or LC-MS/MS. Thus, the question is not the degree to which qPCR can substitute for or is advantageous in comparison with these traditional monitoring approaches, but its usefulness as a complementary tool during risk assessments of cyanobacterial blooms. In all of the studies cited in this review, the qPCR assays and the cyanotoxin quantification were performed in parallel, and this combination will likely continue to be necessary and used in the following years. 

On the other hand, the study of cyanobacteria population dynamics for assessing relative changes in potential toxic cyanobacteria and possible correlations with environmental changes will certainly continue to benefit from qPCR assays for cyanotoxin. In this case, the straight forward nature of this method can be fully explored, by applying qPCR to numerous samples to enable, for example, the monitoring of populations over short time scales and also to investigate spatial variability in freshwater systems.

## Figures and Tables

**Table 1:** Chronological list of studies using qPCR for microcystin genes to estimate the potential toxicity of cyanobacterial blooms. MC, microcystin; PPIA, protein phosphatase inhibition assay; ELISA, Enzyme-linked immunosorbent assay; HPLC, High-performance liquid chromatography. LC-MS/MS, Liquid chromatography-mass spectrometry; Chla, Chlorophyll-a; [MC], Microcystin concentration.

Target Genes	MC Extraction	MC Analysis	[MC] range µg·L^−1^	Correlation between *mcy* genes and [MC].	Correlation between Chla or No. Cells and [MC]	Study Site	Ref.	Year
*mcyE*	Total	HPLC	0–9	Yes	n.d	Lakes Tuusulanjarvi and Hiidenvesi, FI	[51]	2003
*mcyB*	Particulate	HPLC	n.a	n.d	Yes	Lake Wannsee, DE	[49]	2003
*mcyD,* 16S rRNA	Particulate	PPIA	0–15.4	n.d	n.d	Lake Erie, U.S.	[52]	2005
*mcyA*	Total	HPLC	1.3–22.7	n.d	n.d	Lakes Kasumigaura and Kitaura, JP	[53]	2006
*mcyD,* 16S rRNA	Particulate	PPIA	0–2.6	No	Yes	Lake Oneida, NY, U.S.	[57]	2008
*mcyA*, *PG-IGS*	Particulate	PPIA	0–7.4	No	n.d	BNV artificial lake, FR	[58]	2008
*mcyD,* 16S rRNA	Particulate	PPIA	0.1–78.8	Yes	No	Lakes Champlain, Agawam Ronkonkoma and Mill Pond, U.S.	[59]	2009
*mcyA*, 16S rRNA	Total	ELISA	8–8000	Yes	Yes	Hirosawa-No-ike fish pond, JP	[60]	2009
*mcyA*	Particulate	PPIA	0–9	No	n.d	Lake Taihu, CN	[61]	2009
*mcyD*, 16S rRNA	Particulate	PPIA	0-21.7	Yes	Yes	Lake Erie, US	[62]	2009
*mcyD*, 16S rRNA	Particulate	PPIA	0–3.6	No	n.d	San Francisco Bay, U.S.	[63]	2010
*mcyJ*, *PG-IGS*	Dissolved	HPLC	0–0.6	n.d	Yes	Daechung Reservoir, KR	[54]	2010
*mcyB*, *cpcBA*	Particulate	PPIA, LC-MS/MS	1–18	No	Yes	Loire river, FR	[64]	2010
*mcyB*, *PC-IGS*	Particulate	HPLC	0.02–10	n.d	Yes	Lakes Saka, George, Edward, Mburo, Murchison Bay, UG	[65]	2010
*mcyD*	Particulate	ELISA, HPLC	0–4	Yes	n.d	Lake Champlain, CA	[66]	2010
*mcyJ*, *cpcBA*	Particulate	HPLC	0–0.6	Yes	Yes	Daechung Reservoir, KR	[67]	2011
*mcyB*,*A,* 16S rRNA	Particulate	ELISA	2.5–7.0	Yes	No	Tâmega River, PT	[68]	2011
*mcyE*, 16S rRNA	Total	ELISA	0-586	Yes	Yes	Kranji Reservoir, SG	[69]	
*mcyE*	Particulate and dissolved	ELISA	0–25	Yes	Yes	Lake Rotorua, NZ	[70]	2011
*mcyB*, *mcyE,* 16S rRNA	Total	ELISA	0–217	Yes	n.d	Roodeplaat reservoir, ZA	[55]	2012
*mcyB*, *cpcBA*	Particulate	HPLC	10–100	No	Yes	Shallow lake, FR	[71]	2012
*mcyE, cpcBA*	Total	ELISA	0.4–28.7	Yes	Yes	Lake Taihu, CN	[72]	2012
*mcyA, 16S rRNA*	Particulate	LC-MS/MS	0-528	Yes	Yes	Durgakund Pond, Varanasi, IN	[73]	2012
*mcyA,* 16S rRNA	Particulate	HPLC	1.3–48.6	No	n.d	Daechung, Yongdam, Chungju, Soyang, Euam reservoir, KR	[74]	2013
*mcyB*	Particulate	HPLC, ELISA, LC-MS/MS	0–145 *	Yes	n.d	Hauninen reservoir, FI	[75]	2013
*mcyD*	Particulate	ELISA	0–0.5	Yes	n.d	Furnas reservoir, BR	[76]	2013
*mcyE,* 16S rRNA	Particulate	ELISA	0.01–24	Yes	Yes	Missisquoi Bay, CA	[77]	2014
*mcyB*	Particulate	ELISA, LC-MS/MS	0–30.4	Yes	n.d	Aland Islands, FI	[78]	2014
*mcyB,* 16S rRNA	Total	LC-MS/MS	0.02–0.5	No	Yes	Funil reservoir, BR	[79]	2014
*mcyA-E*, *G, J* 16S rRNA	Total	LC-MS/MS	0–0.05	No	No	Macau storage reservoir, CN	[56]	2014
*mcy*A, 16S rRNA	Total	ELISA	n.a	Yes	Yes	Lakes Tai and Yang-cheng, CN	[80]	2014
*mcyD*, *cpcBA*	Particulate	HPLC	0.2–4.2	No	Yes	Lake Taihu, CN	[81]	2014
*mcyD*, 16S rRNA	Particulate and dissolved	HPLC	1–17.6	Yes	Yes	Lake Chaohu, CN	[9]	2014
*mcyE, mcyA,* 16S rRNA	Total	LC-MS/MS	0–66	No	n.d	Lakes Mendota, Monona, Wingra and Kegonsa, U.S.	[82]	2015
*mcyA*, *mcyE*	Particulate and dissolved	ELISA	0–15	Yes	Yes	Vancouver Lake, U.S.	[83]	2015
*mcyB, PC-IGS,*	Total	ELISA	0.3–165	Yes	Yes	Klamath river, U.S.	[10]	2015
*mcyA*	Particulate	ELISA	0–77	Yes	No	Lake Aydat, FR	[11]	2015
*mcyA*, *mcyB*, 16S rRNA	Particulate and dissolved	LC-MS/MS	2.2–38.6	Yes	Yes	Lakshmikund and Sankuldhara, IN	[84]	2015

n.a, not available; n.d, not determined; * mg total MC/g sample dry weight.

**Table 2:** Chronological list of studies using qPCR for cylindrospermopsin genes to estimate the potential toxicity of cyanobacterial blooms. CYL, cylindrospermopsin; ELISA, Enzyme-linked immunosorbent assay; HPLC, High-performance liquid chromatography; LC-MS/MS, Liquid chromatography-mass spectrometry; [CYL], cylindrospermopsin concentration.

Target Genes	CYL Extraction	CYL Analysis	[CYL] range µg·L^−1^	Correlation between *cyr* genes and [CYL]	Study Site	Ref.	Year
*cyrC, rpoC1*	Particulate	LC-MS or MALDI-TOF/MS	n.a	No	Lakes South Australia and Imperial, AU	[86]	2008
*cyrC, rpoC1,* 16S rRNA	Particulate and dissolved	LC–MS/MS	n.a	Yes	Lakes Samsonvale, Somerset and Wivenhoe, AU	[87]	2010
*cyrC,* 16S rRNA*, rpoC1*	Particulate and dissolved	HPLC	0–0.3	n.d	Lake Vela, PT	[88]	2011
*cyrC, rpoC1*	Total	ELISA	0–0.7	n.d	Lake Cheng Kung, TW	[89]	2012
*cyrA*, 16S rRNA	Total	ELISA	0.2–0.6	Yes	Murray river, AU	[8]	2012
*cyrJ*, *rpoC1*	Dissolved	LC-MS/MS	0.1–0.7	Yes	Alange reservoir, ES	[90]	2013
*cyrA,* 16S rRNA	Particulate	LC-MS/MS	n.a	-	North Pine reservoir, AU	[91]	2014
*cyrC, rpcC1*	-	LC-MS/MS	0–1.3	Yes	Macau storage reservoir, MO	[56]	2014

n.a, not available; n.d, not determined.

**Table 3:** Chronological list of studies using qPCR for saxitoxin genes to estimate the potential toxicity of cyanobacterial blooms. STX, saxitoxin; ELISA, Enzyme-linked immunosorbent assay; HPLC, High-performance liquid chromatography; [STX], saxitoxin concentration.

Target Genes	STX Extraction	STX Analysis	[STX] range µg·L^−1^	Correlation between *sxt* genes and [STX]	Study Site	Ref.	Year
*sxtA*, 16S rRNA	Particulate	HPLC	0.08–14.5	Yes	Australian water bodies	[95]	2010
*sxtA*, 16S rRNA	Total	ELISA	0.015–0.023	n.d	Murray River, AU	[8]	2012

n.d, not determined.
